# Rosiglitazone *via* PPARγ-dependent suppression of oxidative stress attenuates endothelial dysfunction in rats fed homocysteine thiolactone

**DOI:** 10.1111/jcmm.12510

**Published:** 2015-02-05

**Authors:** Xu-Hong Yang, Peng Li, Ya-Ling Yin, Jiang-Hua Tu, Wen Dai, Li-Ying Liu, Shuang-Xi Wang

**Affiliations:** a Department of Pharmacology, Pharmaceutical College, Central South UniversityChangsha, China; b College of Pharmacy, Xinxiang Medical UniversityXinxiang, China; c School of Basic Medical Sciences, Xinxiang Medical UniversityXinxiang, China; d The Key Laboratory of Cardiovascular Remodeling and Function Research, Chinese Ministry of Education and Chinese Ministry of Health, Qilu Hospital, School of Medicine, Shandong UniversityJinan, China

**Keywords:** rosiglitazone, homocysteine thiolactone, oxidative stress, endothelial dysfunction, vascular ageing

## Abstract

To explore whether rosiglitazone (RSG), a selective peroxisome proliferator-activated receptor γ (PPARγ) agonist, exerts beneficial effects on endothelial dysfunction induced by homocysteine thiolactone (HTL) and to investigate the potential mechanisms. Incubation of cultured human umbilical vein endothelial cells with HTL (1 mM) for 24 hrs significantly reduced cell viabilities assayed by 3-(4,5-dimethyl-2-thiazolyl)-2,5-diphenyl-2-H-tetrazolium bromide, as well as enhanced productions of reactive oxygen species, activation of nuclear factor kappa B, and increased intercellular cell adhesion molecule-1 secretion. Pre-treatment of cells with RSG (0.001–0.1 mM), pyrollidine dithiocarbamate (PDTC, 0.1 mM) or apocynin (0.1 mM) for 1 hr reversed these effects induced by HTL. Furthermore, co-incubation with GW9662 (0.01 mM) abolished the protective effects of RSG on HTL-treated cells. In *ex vivo* experiments, exposure of isolated aortic rings from. rats to HTL (1 mM) for 1 hr dramatically impaired acetylcholine-induced endothelium-dependent relaxation, reduced release of nitric oxide and activity of superoxide dismutase, and increased malondialdehyde content in aortic tissues. Preincubation of aortic rings with RSG (0.1, 0.3, 1 mM), PDTC or apocynin normalized the disorders induced by HTL. *In vivo* analysis indicated that administration of RSG (20 mg/kg/d) remarkably suppressed oxidative stress and prevented endothelial dysfunction in rats fed HTL (50 mg/kg/d) for 8 weeks. RSG improves endothelial functions in rats fed HTL, which is related to PPARγ-dependent suppression of oxidative stress.

## Introduction

Hyperhomocysteinemia (HHcy) might play a role in the pathogenesis of vascular disorders and is considered as an independent risk factor for atherosclerosis in 1969 1. High levels of homocysteine (HCY) impair endothelial function and cause endothelial damage in humans as well as in animal models 2,3, indicating that the endothelial monolayer is very sensitive to changes in plasma HCY levels. The adverse effects of HCY on endothelial function involve a decrease in nitric oxide bioavailability 4. One of the possible mechanisms involved with the effects of HCY is the excessive generations of hydrogen peroxide, superoxide anion and reactive oxygen species (ROS), which increases the oxidative degradation of nitric oxide. Normalization of redox state in HHcy might be an approach to prevent HHcy-induced vascular disease.

Peroxisome proliferator-activated receptors (PPARs) are ligand-activated transcription factors belonging to the nuclear receptor superfamily, which are present in three isoforms as α, β and γ 5,6. Earlier studies have shown that disruption or down-regulation of PPARγ resulted in vascular endothelial dysfunction 7. PPARγ, in particular, has been implicated in the pathology of several diseases including obesity, diabetes, atherosclerosis and cancer 6. However, the role of PPARγ signaling in HHcy-induced endothelial dysfunction needs further investigations.

As an agonist of PPARγ, rosiglitazone (RSG) is an oral anti-diabetic drug that has been shown to improve glycemic control by reducing insulin resistance 8. Clinical observations have indicated that RSG improves vascular function in diabetic patients 9,10 or non-diabetic populations with insulin resistance 11,12, suggesting a possible beneficial effect on cardiovascular prognosis. Moreover, RSG exerts beneficial effects to prevent the detrimental effects of HHcy on vascular dementia, cardiac hypertrophy and intimal hyperplasia in rats 13. On the basis of the mentioned studies, we tested the hypothesis that RSG may produce protective effects on HHcy-induced endothelial dysfunction *via* suppression of oxidative stress. Here, we reported that pharmacological activation of PPARγ by RSG improves endothelial function in rats fed homocysteine thiolactone (HTL), which is one of the most reactive forms of HCY metabolites 14.

## Materials and methods

### Materials

Rosiglitazone (Cat: R2408), HTL (Cat: 53530), GW9662 (Cat: M6191), pyrollidine dithiocarbamate (PDTC, Cat: P8765), dihydroethidium (DHE, Cat: D7008), 4′,6-diamidino-2-phenylindole (DAPI, Cat: 32670), apocynin (Cat: A10809), acetylcholine (Ach, Cat: A6625), sodium nitroprusside (SNP, Cat: 71778) and phenylephrine (PE, Cat: P1240000) were purchased from Sigma Company (St. Louis, MO, USA). Primary antibodies against p65 (Cat: 8008), phosphor-p65 (Cat: 166748), p47 (Cat: 365215), p67 (Cat: 374510) and GAPDH (Cat: 365062) were from Santa Cruz Company (Santa Cruz, CA, USA).

### Animals

Male Sprague–Dawley rats (8 ± 2 weeks old, 180 ± 20 g) were purchased from the Center of Experiment Animals, Central South University (Changsha, China). Rats were housed in temperature-controlled cages with a 12-hr light–dark cycle. The animal protocol was reviewed and approved by the University of Central South Animal Care and Use Committee.

### Cell culture

Human umbilical vascular endothelial cells (HUVECs) from American Type Culture Collection were grown in endothelial cells basal medium (Clonetics Inc., Walkersville, MD, USA) supplemented with 2% foetal bovine serum, penicillin (100 U/ml), and streptomycin (100 μg/ml). In all experiments, cells were between passages 3 and 8. All cells were incubated at 37°C in a humidified atmosphere of 5% CO_2_ and 95% air. Cells were grown to 70–80% confluence before being treated with different agents.

### Organ chamber

Organ chamber study was performed as described previously 15. Rats were killed under anaesthesia by intravenous injection with pentobarbital sodium (30 mg/kg). The descending aorta isolated by removing the adhering perivascular tissue carefully was cut into rings (3–4 mm in length). Aortic rings were suspended and mounted to organ chamber by using two stainless hooks. The rings were placed in organ baths filled with Kreb's buffer of the following compositions (in mM): NaCl, 118.3; KCl, 4.7; MgSO_4_, 0.6; KH_2_PO_4_, 1.2; CaCl_2_, 2.5; NaHCO_3_, 25.0; EDTA, 0.026; pH 7.4 at 37°C and gassed with 95% O_2_ plus 5% CO_2_, under a tension of 2 g, for 90-min. equilibration period. During this period, the Kreb's solution was changed every 15 min. After the equilibration, aortic rings were challenged with 60 mM KCl. After washing and another 30-min. equilibration period, contractile response was elicited by PE (1 μM). At the plateau of contraction, accumulative Ach (0.01, 0.03, 0.1, 0.3, 1, 3 μM) or SNP (0.01, 0.03, 0.1, 0.3, 1, 3, 10 μM) was added into the organ bath to induce endothelium-dependent or -independent relaxation.

For *ex vivo* experiments, the rings were contracted by PE (1 μM) and then dilated with cumulative concentrations of Ach (0.01–3 μM) to assess the integrity of the endothelium. The ring which the maximal relaxation induced by Ach (3 μM) is over 80% was considered to have intact endothelium and were used in the following studies. Then, the rings were pre-treated with RSG (0.1, 0.3, 1 mM), PDTC (0.1 mM) or apocynin (0.1 mM) for 30 min. followed by addition of HTL (1 mM) for 90 min. After washing, Ach-induced endothelium-dependent relaxation and SNP-induced endothelium-independent relaxation were assayed respectively. At the end of experiments, the aortic rings were collected and kept in liquid nitrogen for measurements of nitric oxide, malondialdehyde (MDA) and superoxide dismutase (SOD) activity after homogenized.

### Measurements of nitric oxide, MDA and SOD activity

The contents of nitric oxide content (Cat: 10009419), MDA content (Cat: 700870), and SOD activity (Cat: 706002) in aortic tissues or serum were assayed by using commercial kits from Cayman Company (Ann Arbor, MI, USA). Soluble intercellular adhesion molecule 1 (sICAM-1) commercial kit (EIA-SK00250-02) was from Aviscera Bioscience, Santa Clara, CA, USA.

### Evaluation of cell viability

Cell viability was assayed by using 3-(4,5-dimethyl-2-thiazolyl)-2,5-diphenyl-2-H- tetrazolium bromide (MTT) as described previously 16. Cells were seeded into 96-well plate at the density of 10,000/ml and incubated for 24 hrs. After treatment, 10 μl MTT (5 mg/ml) was added into cultured medium in each well for 2–4 hrs until purple precipitate is visible. After removal of culture medium, 75 μl dimethyl sulfoxide was added to each well and leave the cells at room temperature in the dark for 2 hrs. The absorbance at 570 nm was recorded.

### Detection of ROS

Reactive oxygen species productions in cultured cells were assayed by measuring the DHE fluorescence, combined with high performance liquid chromatography (HPLC) with minor modifications 17. Briefly, cells were incubated with DHE (10 μM) for 30 min., washed, harvested and homogenized and subjected to methanol extraction. HPLC was performed by using a C-18 column (mobile phase: gradient of acetonitrile and 0.1% trifluoroacetic acid) to separate and quantify oxyethidium (product of DHE and O_2_^−^) and ethidium (a product of DHE auto-oxidation). O_2_^−^ production was determined by conversion of DHE into oxyethidine.

### Reverse transcription polymerase chain reaction

Total cellular RNA was isolated using a Qiagen reagent (Dusseldorf, Germany) and reverse transcribed to cDNA with specific antisense primers using the ThermoScript reverse transcription polymerase chain reaction (RT-PCR) System protocol (Invitrogen, Shanghai, China). Samples (2 μl) of reverse transcribed product were PCR-amplified in a total volume of 25 μl with 10 pM each of forward and reverse primers of PPARγ (Forward: AGCCCTTTGGTGACTTTATGGA; Reverse: TCCTCAATGGGCTTCACGTT). GAPDH (Forward: CAAGGTCATCCATGACAAC TTTG; Reverse: GTCCACCACCCTGTTGCTGTAG) served as a control.

### Immunofluorescence

Nuclear factor kappa B (NF-κB) activation was determined by using immunofluorescence (IFC) as described previously 18. In brief, cultured cells grown on sterile glass cover slips were rinsed by cold PBS and then fixed by incubation with 10% formalin in PBS for 10 min. Block cells by 5% BSA for 30 min. Incubate cells with primary antibody for 1 hr at room temperature or overnight at 4°C. After washing, incubate with fluorescence- conjugated secondary antibody for 45 min. Take picture in fluorescence microscope.

### Immunohistochemistry

As described previously 19, the thoracic aorta was fixed in 4% paraformaldehyde overnight, and then processed, embedded in paraffin and sectioned at 4 μm. The deparaffinized, rehydrated section from thoracic aorta and cryosections from aortic root (5 μm) were microwaved in citrate buffer for antigen retrieval. Sections were incubated in endogenous peroxidase (DAKO, Via Real, Carpinteria, CA) and protein block buffer, and then with primary antibodies indicated overnight at 4°C. Slides were rinsed with washing buffer and incubated with labelled polymer-horseradish peroxidase secondary antibodies followed by DAB^+^ chromogen detection (DAKO). After final washes, sections were counterstained with haematoxylin. All positive staining was confirmed by ensuring that no staining occurred under the same conditions with the use of non-immune rabbit or mouse control IgG.

### Determinations of plasma HTL and HCY levels

The determination of HTL has been described previously 20. Plasma levels of HCY were measured by highly selective analytical methods like HPLC combined with fluorescence detection as described previously 21.

### Transfection of siRNA into cells

Transient transfection of siRNA was carried out according to Santa Cruz's protocol as described by us 22. Briefly, the siRNAs were dissolved in siRNA buffer (20 mM KCl; 6 mM HEPES, pH 7.5; 0.2 mM MgCl_2_) to prepare a 10 μM stock solution. Cells grown in 6-well plates were transfected with siRNA in transfection medium containing liposomal transfection reagent (Lipofectamine RNAiMax, Invitrogen). For each transfection, 100 μl transfection medium containing 4 μl siRNA stock solution was gently mixed with 100 μl transfection medium containing 4 μl transfection reagent. After a 30-min. incubation at room temperature, siRNA-lipid complexes were added to the cells in 1.0 ml transfection medium, and cells were incubated with this mixture for 6 hrs at 37°C. The transfection medium was then replaced with normal medium, and cells were cultured for 48 hrs.

### Western blot analysis

As described previously 23, aortic tissues were homogenized on ice in cell-lysis buffer (20 mM Tris-HCl, pH 7.5, 150 mM NaCl, 1 mM Na_2_EDTA, 1 mM EGTA, 1% Triton, 2.5 mM sodium pyrophosphate, 1 mM beta-glycerophosphate, 1 mM Na_3_VO_4_, 1 μg/ml leupeptin) and 1 mM PMSF. Cell was lysated with cell-lysis buffer. The protein content was assayed by BCA protein assay reagent (Pierce, Rockford, IL, USA). 20 μg proteins were loaded to SDS-PAGE and then transferred to membrane. Membrane was incubated with a 1:1000 dilution of primary antibody, followed by a 1:2000 dilution of horseradish peroxidase-conjugated secondary antibody. Protein bands were visualized by electrochemiluminescence (GE Healthcare, Pittsburgh, PA, USA). The intensity (area × density) of the individual bands on Western blots was measured by densitometry (model GS-700, Imaging Densitometer; Bio-Rad, Hercules, CA, USA). The background was subtracted from the calculated area. We used control as 100%.

### Statistical analysis

All quantitative results are expressed as mean ± SEM. The vessel responses to Ach or SNP are expressed as percentages of pre-contractions and these data were analysed using a two-way anova followed by Bonferroni corrections. One-way anova was used to compare multiple groups followed by Newman–Keuls student *t*-tests. Statistical analysis was conducted using IBM SPSS statistics 20.0 (IBM Corp., Armonk, NY, USA) and *P* < 0.05 were considered as statistical significance.

## Results

### RSG dose-dependently normalizes cell viabilities in HTL-treated endothelial cells

**Fig 1:**
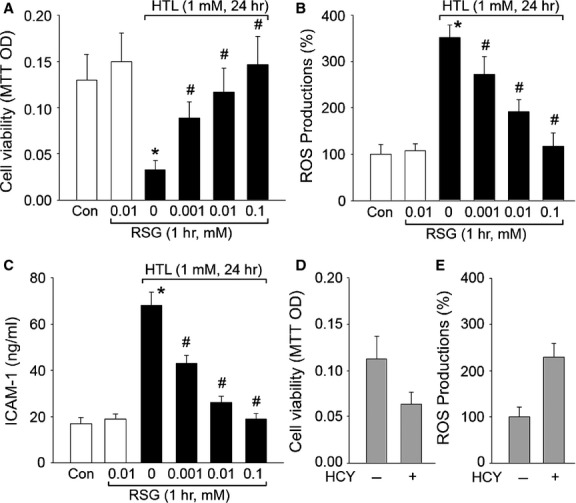
Rosiglitazone dose-dependently suppresses HTL-induced oxidative stress and improves cell viabilities in cultured HUVECs. Cultured HUVECs were pre-treated with rosiglitazone (RSG, 0.001–1 mM) for 1 hr and then incubated with homocysteine thiolactone (HTL, 1 mM) for 24 hrs. (A) Cell viability was measured by MTT. (B) Intracellular ROS productions were detected by DHE/HPLC. (C) The concentration of soluble intercellular adhesion molecule 1 (sICAM-1) in culture medium was assayed by ELISA. All data were expressed as mean ± SEM. *N* is 3 in each group. *P < 0.05 *versus* Con, ^#^*P* < 0.05 *versus* HTL alone. (D and E) Cultured HUVECs were treated with homocysteine (HCY, 1 mM) for 24 hrs. (D) Cell viability and (E) intracellular ROS productions were measured by MTT and DHE/HPLC respectively. Data were expressed as mean ± SEM. *N* is 3 in each group. *P < 0.05 *versus* Control.

Homocysteine occurs in human blood plasma in several forms. The most reactive form is HTL, which is a cyclic thioester representing up to 0.29% of total plasma HCY 14. HTL reacts with proteins by acylation of free basic amino groups. In particular, the epsilon-amino group of lysine residues forms adducts and induces structural and functional changes in plasma proteins. Thus, we firstly investigated whether HTL affected cell viabilities in cultured HUVECs. As shown in Figure1A, incubation of HUVECs with HTL (1 mM) for 24 hrs dramatically reduced cell viabilities detected by MTT. Although RSG alone did not affect endothelial cell viabilities, it dose-dependently restored cell viabilities reduced by HTL (Fig.1A). These data indicate that RSG normalizes cell viabilities in HTL-treated endothelial cells.

### RSG dose-dependently suppresses HTL-induced oxidative stress in cultured endothelia cells

Oxidative stress is a common mechanism of risk factors to induce endothelial dysfunction in cardiovascular diseases 24. We have been suggested that RSG maintains the normal phenotypes of vascular endothelial cells *via* suppression of oxidative stress. To confirm this hypothesis, we measured intracellular ROS productions and secretion of sICAM-1 in endothelial cells. As expected in Figure1B and C, incubation of HUVECs with HTL (1 mM) for 24 hrs remarkably increased ROS productions and secretion of sICAM-1 in cultured endothelial cells. However, preincubation of endothelial cells with RSG inhibited the enhancements of ROS productions and secretion of sICAM-1 induced by HTL in dose-dependent manner. Taken together, these results indicate that RSG preserves cell viability, which is reduced by HTL, possibly by suppression of oxidative stress.

Homocysteine thiolactone is also able to directly incorporate into proteins *via* homocysteinylation altering protein structure and function 25. To exclude this possibility, we tested another chemical form of HCY on cell viabilities and oxidative stress. We found HCY also damaged endothelial cell functions and increased ROS productions (Fig.1D and E) in cultured cells, further supporting that HTL impaired endothelial cell functions through oxidative stress.

### RSG *via* PPARγ activation protects the functions of HTL-treated cells

**Fig 2:**
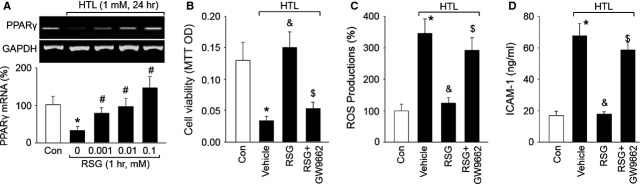
Inhibition of PPARγ abolished rosiglitazone-suppressed oxidative stress induced by HTL in endothelial cells. (A) Cultured HUVECs were pre-treated with rosiglitazone (RSG, 0.001–1 mM) for 1 hr and then incubated with homocysteine thiolactone (HTL, 1 mM) for 24 hrs. The expression of PPARγ mRNA was examined by RT-PCR. All data were expressed as mean ± SEM. The picture is a representative picture from three independent experiments. *P < 0.05 *versus* Con, ^#^*P* < 0.05 *versus* HTL alone. (B–D) Cultured HUVECs were pre-treated with rosiglitazone (RSG, 1 mM) for 1 hr and then incubated with homocysteine thiolactone (HTL, 1 mM) for 24 hrs in presence of GW9662 (0.01 mM). The expression of PPARγ mRNA was examined by RT-PCR. Cell was subjected to assay (B) Cell viability by MTT, (C) Intracellular ROS productions by DHE fluorescence and (D) The concentration of sICAM-1 in culture medium by ELISA. All data were expressed as mean ± SEM. N is 3 in each group. *P < 0.05 *versus* Con, ^&^*P* < 0.05 *versus* HTL alone, ^$^*P* < 0.05 *versus* HTL+RSG.

We next determined whether RSG provided beneficial effects in endothelial cells *via* activation of PAPRγ. Thus, the gene expression of PPARγ was assayed by RT-PCR. In Figure2A, treatment of cells with HTL significantly reduced PPARγ mRNA level. This is consistent with other group's observations on animal models 26. Furthermore, RSG dose-dependently reversed the gene expressions of PPARγ (Fig.2A). Then we disrupted PPARγ signaling by using GW9662, which is a specific antagonist of PPARγ 27. As expected, inhibition of PPARγ by GW9662 significantly abolished the protective effects of RSG on the improvement of cell viabilities (Fig.2B), reductions in ROS productions (Fig.2C) and secretion of sICAM-1 (Fig.2D). Taken together, these data indicate that RSG suppresses oxidative stress and protects cell viability *via* PPARγ activation.

### Inhibition of NF-κB or NAD(P)H oxidase reverses HTL-induced oxidative stress in cells

**Fig 3:**
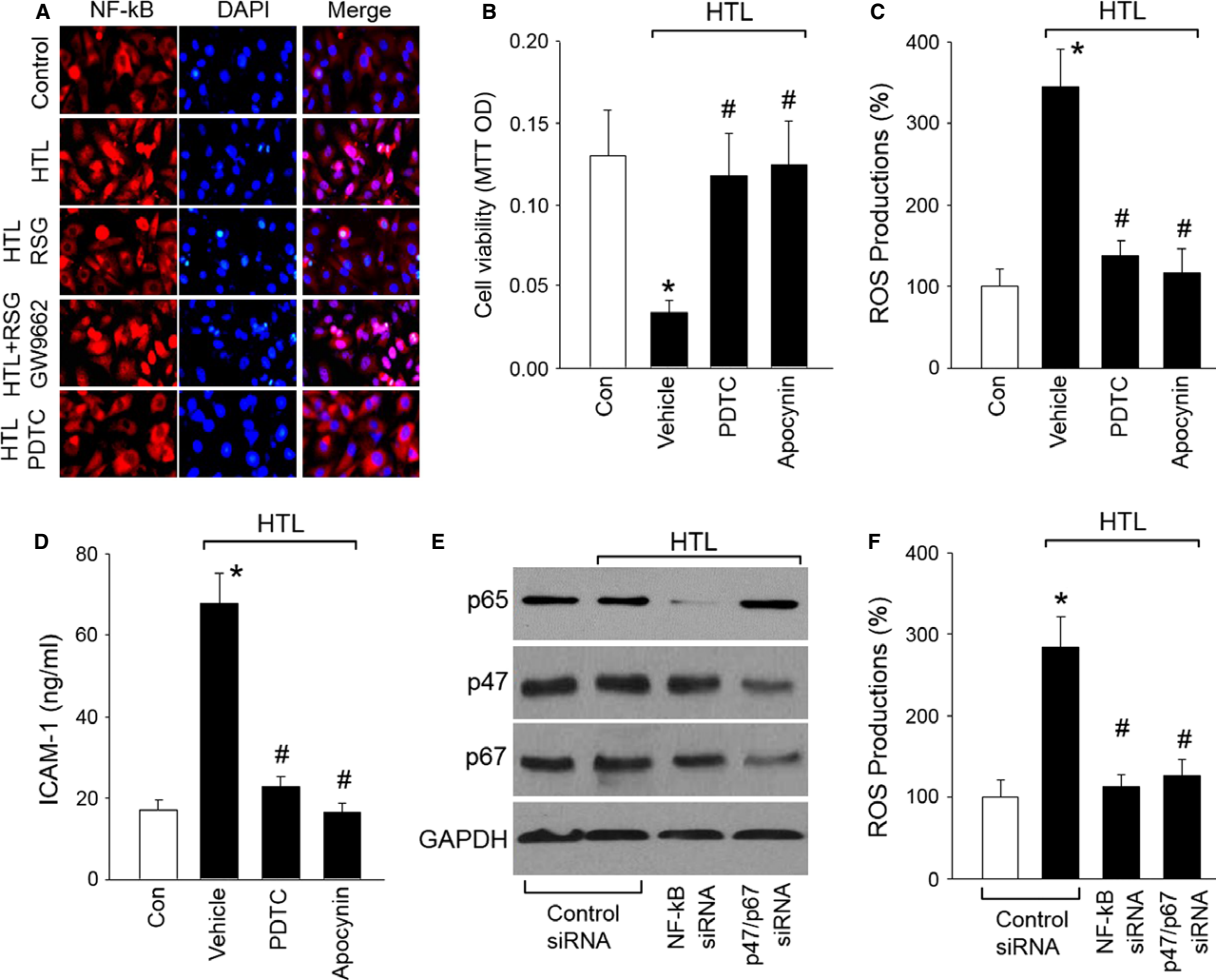
Rosiglitazone *via* PPARγ inhibits NF-κB to suppress oxidative stress and protects cell functions in endothelial cells. (A) Cultured HUVECs were pre-treated with 1 mM RSG, 1 mM RSG plus 0.01 mM GW9662 or 0.1 mM PDTC for 1 hr and then incubated with homocysteine thiolactone (HTL, 1 mM) for 24 hrs. IHC fluorescence was used to detect NF-κB activity by staining cells with primary anti-NF-κB antibody. Red, NF-κB; Blue, DAPI (nucleus). The picture is a representative picture from three independent experiments. (B–D) Cultured HUVECs were pre-treated with 0.1 mM PDTC or apocynin (0.1 mM) for 1 hr and then incubated with homocysteine thiolactone (HTL, 1 mM) for 24 hrs. Cell was subjected to assay (B) Cell viability by MTT, (C) Intracellular ROS productions by DHE fluorescence, and (D) The concentration of sICAM-1 in culture medium by ELISA. All data were expressed as mean ± SEM. N is 3 in each group. *P < 0.05 *versus* Con, ^#^*P* < 0.05 *versus* HTL alone. (E and F) Cultured HUVECs were transfected with control siRNA, NF-κB (p65) siRNA and NAD(P)H oxidase (p47/p67) siRNA for 48 hrs. Then cells were treated with HTL (1 mM) for 24 hrs. (E) Protein levels of NF-κB p65, and NAD(P)H oxidase (p47 and p67) in total cell lysates and (F) intracellular ROS productions were measured by Western blot and DHE/HPLC respectively. Data were expressed as mean ± SEM. N is 3 in each group. *P < 0.05 *versus* Control siRNA alone. ^#^*P* < 0.05 *versus* Control siRNA plus HTL.

Nuclear factor kappa B is anchored and inactivated in the cytoplasm by association with IκBα 28. NF-κB-dependent up-regulation of NAD(P)H oxidase contributes to oxidative stress in endothelial cells 17. We next investigated whether RSG-suppressed NF-κB activity in HTL-treated cells by staining NF-κB p65 in cultured cells with the primary antibody. In Figure3A, IFC analysis indicated that treatment of cells with HTL significantly increased NF-κB location in nucleus, which was reversed by RSG. However, GW9662 abolished RSG-induced inhibition of NF-κB. PDTC, as a NF-κB inhibitor 29, also inhibited HTL-induced NF-κB activation.

We then determined if either NF-κB or NAD(P)H oxidase is involved in HTL-induced oxidative stress in endothelial cells. We blocked NF-κB by specific inhibitor of PDTC or NAD(P)H oxidase by apocynin 17 in HTL-treated cells. As expected, preincubation of endothelial cells with PDTC or apocycnin for 30 min. prior to HTL significantly limited the impairment of cell viabilities (Fig.3B), enhancements of ROS productions (Fig.3C) and secretion of sICAM-1 (Fig.3D) induced by HTL.

To exclude the possibility that these results were because of non-specific effects of PDTC or apocynin treatment, we repeated these experiments with cells transfected with specific siRNA. As shown in Figure3E and F, HTL still effectively increased ROS productions in HUVECs transfected with control siRNA, it failed to induce oxidative stress in HUVECs when protein expressions of NF-κB (p65) and NAD(P)H oxidase subunits (p47 and p67) were suppressed by siRNA. Taken together, these results suggest that RSG may inhibit NF-κB to down-regulate NAD(P)H oxidase, resulting in the suppression of oxidative stress in endothelial cells.

### RSG preserves endothelium-dependent relaxation impaired by HTL in isolated rats aortic rings

**Fig 4:**
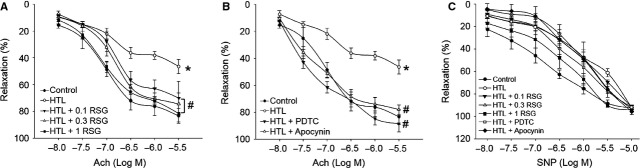
Rosiglitazone attenuates HTL-impaired endothelium-dependent relaxation in rat aortas. (A) The isolated rat aortic rings were exposed to HTL (1 mM) for 1 hr after preincubation with rosiglitazone (RSG, 0.1–1 mM) for 30 min. The endothelium-dependent relaxation induced by acetylcholine (Ach) was assayed by organ chamber. (B) The isolated rat aortic rings were exposed to HTL (1 mM) for 1 hr after preincubation with 0.1 mM PDTC or apocynin (0.1 mM) for 30 min. The endothelium-dependent relaxation induced by acetylcholine (Ach) was assayed by organ chamber. All data were expressed as mean ± SEM. N is 5 in each group. *P < 0.05 *versus* Con, ^#^*P* < 0.05 *versus* HTL alone. (C) Endothelium-independent relaxation was assayed in A and B by using sodium nitroprusside (SNP).

We then performed *ex vivo* experiments to test whether RSG protected vascular endothelial functions by incubating isolated rats aortic rings with HTL. Exposure of aortic rings to HTL dramatically impaired Ach-induced endothelium-dependent relaxation (Fig.4A). This is consistent with our previous study 30. Similar to *in vitro* observations from cultured cells, RSG dose-dependently reversed Ach-induced endothelium-dependent relaxation in aortic rings incubated with HTL (Fig.4A), suggesting that RSG functions as a protector on vascular endothelium.

### Inhibition of NF-κB or NAD(P)H oxidase attenuates HTL-induced impairment of endothelium-dependent relaxation *ex vivo*

Then the inhibition of NF-κB or NAD(P)H oxidase on HTL-impaired endothelial function was determined. As shown in Figure4B, preincubation of rats aortic rings with PDTC or apocycnin improved Ach-induced endothelium-dependent relaxation reduced by HTL, indicating HTL *via* NF-κB/NAD(P)H oxidase pathway damaged the function of vascular endothelium. In addition, SNP-induced endothelium-independent relaxation was not altered in all groups (Fig.4C), indicating that HTL damaged the function of vascular endothelium, but not vascular smooth muscle.

### RSG preserves redox state in HTL-disturbed aortas

**Fig 5:**
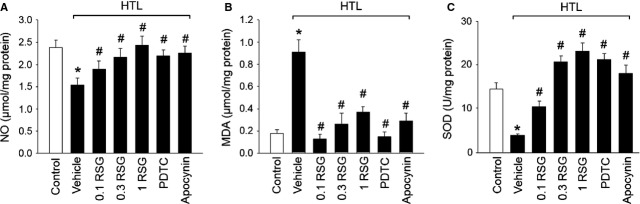
HTL *via* PPARγ/NF-κB/NADPH oxidase induces oxidative stress in isolated rat aortas. The isolated rat aortic rings were exposed to HTL (1 mM) for 1 hr after preincubation with rosiglitazone (RSG, 0.1–1 mM), PDTC (0.1 mM) or apocynin (0.1 mM) for 30 min. Homogenates of aortic tissues were subjected to assay (A) nitric oxide productions by Griess method, (B) MDA content by TBA method and (C) SOD activity by the spectrophotometric method. All data were expressed as mean ± SEM. N is 5 in each group. *P < 0.05 *versus* Con, ^#^*P* < 0.05 *versus* HTL alone.

Decreased nitric oxide bioavailability, which is because of decreased nitric oxide production or aberrant conversion of nitric oxide to ONOO^−^ by ROS, contributes to impairment of Ach-induced endothelium-dependent relaxation in cardiovascular system 31. We then examined whether HTL maintains normal redox state in rat isolated aortic rings. We found that HTL dramatically decreased nitric oxide production (Fig.5A) and increased the content of MDA (Fig.5B), which is formed when ROS reacts with polyunsaturated fatty acid chain in membrane lipids 32. Also, HTL reduced the activity of SOD (Fig.5C), which is an important anti-oxidative enzyme to catalyse the dismutation of O_2_^−^ into H_2_O_2_ 33. However, pre-treatment of cells with RSG, PDTC or apocynin rescued the abnormalities of nitric oxide (Fig.5A), MDA (Fig.5B), and SOD activity (Fig.5C) in HTL-incubated aortas. Taking all data together, it indicates that RSG *via* PPARγ/NF-κB/NAD(P)H oxidase preserves the normal balance of the anti-oxidant systems which are disturbed by HTL.

### Administration of RSG improves endothelial function in rats fed HTL

**Fig 6:**
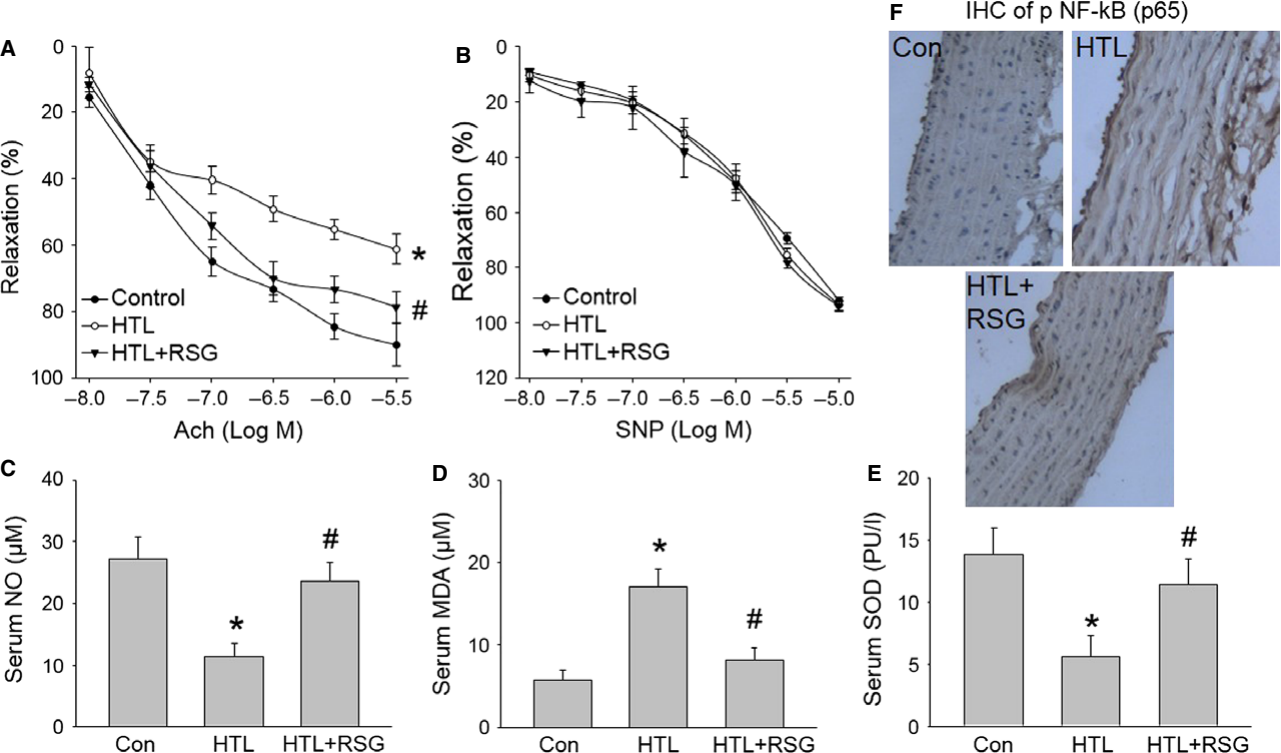
Rosiglitazone suppresses oxidative stress and improves endothelial functions in rats fed HTL *in vivo*. The rats were intragastric gavaged HTL (50 mg/kg/d) and received administration of rosiglitazone (RSG, 20 mg/kg/d) for 8 weeks. At the end of experiments, rats were killed under anaesthesia. Artery from descending aorta was subjected to assay (A) endothelium-dependent relaxation induced by acetylcholine (Ach) and (B) endothelium-independent relaxation induced by sodium nitroprusside (SNP) in organ chamber. Blood was collected to assay (C) serum nitric oxide level by Griess method, (D) serum MDA content by TBA method and (E) SOD activity by the spectrophotometric method. All data were expressed as mean ± SEM. Five to ten rats in each group. *P < 0.05 *versus* Con, ^#^*P* < 0.05 *versus* HTL alone. (F) Immunohistochemical analysis of active NF-κB in arteries by using phospho-p65 antibody. The picture (×40) is a representative picture from five independent experiments.

We then performed *in vivo* experiments to confirm whether RSG improves vascular endothelial functions in rats with HHcy. HCY circulates as different species, mostly protein bound, and approximately 1% as its reduced form and the cyclic thioester HTL. Despite the level of plasma thiolactone being markedly low, detrimental effects of HCY are related to the high reactivity of HTL 34. We fed rats with HTL (50 mg/kg/day) for 8 weeks to mimic the model of HHcy-induced endothelial dysfunction. Treatment of rats with HTL dramatically increased plasma HTL levels, compared to control mice (7.38 ± 1.94 *versus* 2.91 ± 1.08 μM, *P* < 0.01). The plasma level of HCY (7.28 ± 2.12 *versus* 5.64 ± 1.73 μM, *P* < 0.05) was also slightly increased by treatment of HTL. These indexes indicated that supplementation of HTL in diet induced high HCY and HTL model in rats. As shown in Figure6A, HTL significantly inhibited Ach-induced vascular relaxation. Importantly, the reduction in Ach-induced vascular relaxation was reversed by administration of RSG. Both HTL and RSG did not change SNP-induced vessel relaxation (Fig.6B). Also, RSG did not lower plasma levels of HTL and HCY. All data suggest that the improvement of RSG on vascular bioactivity in HTL-fed rats is because of the maintenance of endothelial function, but does not depend upon the plasma HTL and HCY levels.

### Administration of RSG suppresses oxidative stress in rats fed HTL

We finally determined whether RSG preserves the normal redox state in HTL-fed rats. As expected, HTL induced the alternations, such as decreased serum nitric oxide level (Fig.6C), increased serum MDA level (Fig.6D), and decreased serum SOD activity (Fig.6E) in blood from rats. All these defects induced by HTL were normalized by administration of RSG. As expected, RSG also suppressed active NF-κB levels of phosphorylated p65 in aortic tissues assayed by immunohistochemistry (IHC; Fig.6F). The data indicate that the protective effects of RSG may be related to suppression of oxidative stress.

## Discussion

This study demonstrates that HTL *in vitro* or *in vivo* causes accelerated oxidative stress and endothelial dysfunction, all of which are abrogated by RSG, an agonist of PPARγ. Mechanistically, the protective effect of RSG on vascular function is attributable to PPARγ activation, resulting in suppressions of NF-κB and NAD(P)H oxidase. These findings suggest that PPARγ-dependent suppression of NF-κB and consequent down-regulation of NAD(P)H oxidase reduces oxidative stress and, in this way, RSG normalizes the redox state in endothelial cells.

The major finding in this paper is that RSG prevents HTL-induced endothelial dysfunction *in vitro* and *in vivo*. Recent studies have found that the conversion of HCY into HTL plays a critical role in the progress of cardiovascular diseases in patients with HHcy. In this present study, we used HTL to treat isolated aortic ring *ex vivo* or rats *in vivo*, by which both impaired Ach-induced endothelium-dependent relaxation, consistent with our previous study 30. This observation strongly support that detrimental effects of HCY are related to the high reactivity of HTL, though the level of plasma thiolactone is very low. Most importantly, HTL-induced endothelial dysfunction both *ex vivo* and *in vivo* was reversed by RSG in a dose-dependent manner. Collectively, our results suggest that RSG functions as a protector of endothelial cells. This discovery is also supported by several published studies done in cultured endothelial cells 35, which have shown that RSG protects endothelial function. However, a recent study on human reported that RSG caused endothelial dysfunction in normal volunteers. Of course, the reason for this discrepancy between healthy and HHcy needs further investigations.

Another important discovery of this study is that activation of PPARγ by RSG *via* suppression of NF-κB/NAD(P)H oxidase pathway maintains the normal redox state in cells. Under physiological condition, the major source of ROS in endothelial cells is mitochondrial respiratory chain. After generation, ROS is removed rapidly by SOD, paraoxonase and catalase. Once endothelial cell is activated, ROS is produced from other sources, such as NAD(P)H oxidase, in which excessive ROS inactivates nitric oxide by formation of ONOO^−^ and decreases endothelium-dependent relaxation^17^. In this present study, we have identified NAD(P)H oxidase as the main source of ROS in HTL-treated endothelial cells and have shown that RSG *via* PPARγ-dependent suppression of NF-κB to inhibit NAD(P)H oxidase-derived ROS productions. Our investigation is supported by Wagner *et al*. in which they found PPARγ activation reduced expression of Nox1 and Nox4 in mice 36. From our data, we speculate that the mechanism of RSG-suppressed NAD(P)H oxidase in endothelial cells might be related to inhibition of NF-κB *via* PPARγ. Indeed, it has been reported that PPARγ can regulate NF-κB activation 37 and the subunits of NAD(P)H oxidase are NF-κB-targeted genes in vascular cells 17. Therefore, RSG normalizes the redox state in vascular endothelial cells and is considered as a drug to improve endothelial function in the preventions of HHcy patients.

Homocysteine thiolactone is a physiological metabolite of HCY. However, it has been proven to be more cytotoxic than HCY. Moreover, the mechanisms of HCY-induced endothelial injury are more complicated than HTL, which are related to cystathionine β-Synthase, VitB12 and folic acid. Therefore, we used HTL to induce endothelial dysfunction in rats because HTL is reported to be the main factor in HCY-induced oxidative stress in cells 25. Another issue is that HTL can incorporate into proteins directly *via* homocysteinylation altering protein structure and function. In this study, we have detected the effects of other chemical forms, such as HCY, on endothelial cell functions. Similar to HTL, HCY itself triggered oxidative stress in cell. This indicates that HTL *via* oxidative stress damages endothelium, which is independent of homocysteinylation.

In summary, these studies support a novel function of RSG which activates PPARγ to suppress NF-κB-dependent NAD(P)H oxidase. This, in turn, inhibits oxidative stress in endothelial cells, leading to improvement of endothelial function. The findings that RSG attenuates endothelial dysfunction induced by HTL through suppression of oxidative stress may have broad applications for cardiovascular diseases, since endothelial dysfunction is a common character at the beginning and in the progress in a number of vascular diseases, including atherosclerosis, diabetes and vascular ageing or stiffness.
